# Endoscopic diagnosis and management of eosinophilic esophagitis

**DOI:** 10.1002/deo2.70063

**Published:** 2025-01-16

**Authors:** Fumio Tanaka, Akinari Sawada, Sayaka Tanaka, Kenichi Kohashi, Yasuhiro Fujiwara

**Affiliations:** ^1^ Department of Gastroenterology Graduate School of Medicine Osaka Metropolitan University Osaka Japan; ^2^ Department of Pathology Graduate School of Medicine Osaka Metropolitan University Osaka Japan

**Keywords:** diagnosis, endoscopy, eosinophilic esophagitis, eosinophils, therapeutics

## Abstract

Eosinophilic esophagitis is a chronic allergic inflammatory disease, and its incidence and prevalence have recently increased. Eosinophilic esophagitis has not become a rare disease; thus, knowledge for diagnosing it is needed in current clinical practice. The adequate management of endoscopic procedures is particularly important for the diagnosis and evaluation of inflammatory activity and therapeutic responses. The therapeutic options for eosinophilic esophagitis include anti‐acid drugs such as proton pump inhibitors, potassium‐competitive acid blockers, swallowed topical corticosteroids, biologics, and dietary elimination therapies. Moreover, endoscopic esophageal dilation is a therapeutic option for fibrotic strictures due to eosinophilic esophagitis to improve obstructive symptoms, such as dysphagia and food impaction. In this review, we describe the endoscopic characteristics of eosinophilic esophagitis, including an endoscopic reference score and an optimal biopsy protocol to diagnose and evaluate the therapeutic response. We also describe a current therapeutic management of eosinophilic esophagitis.

## INTRODUCTION

**FIGURE 1:**
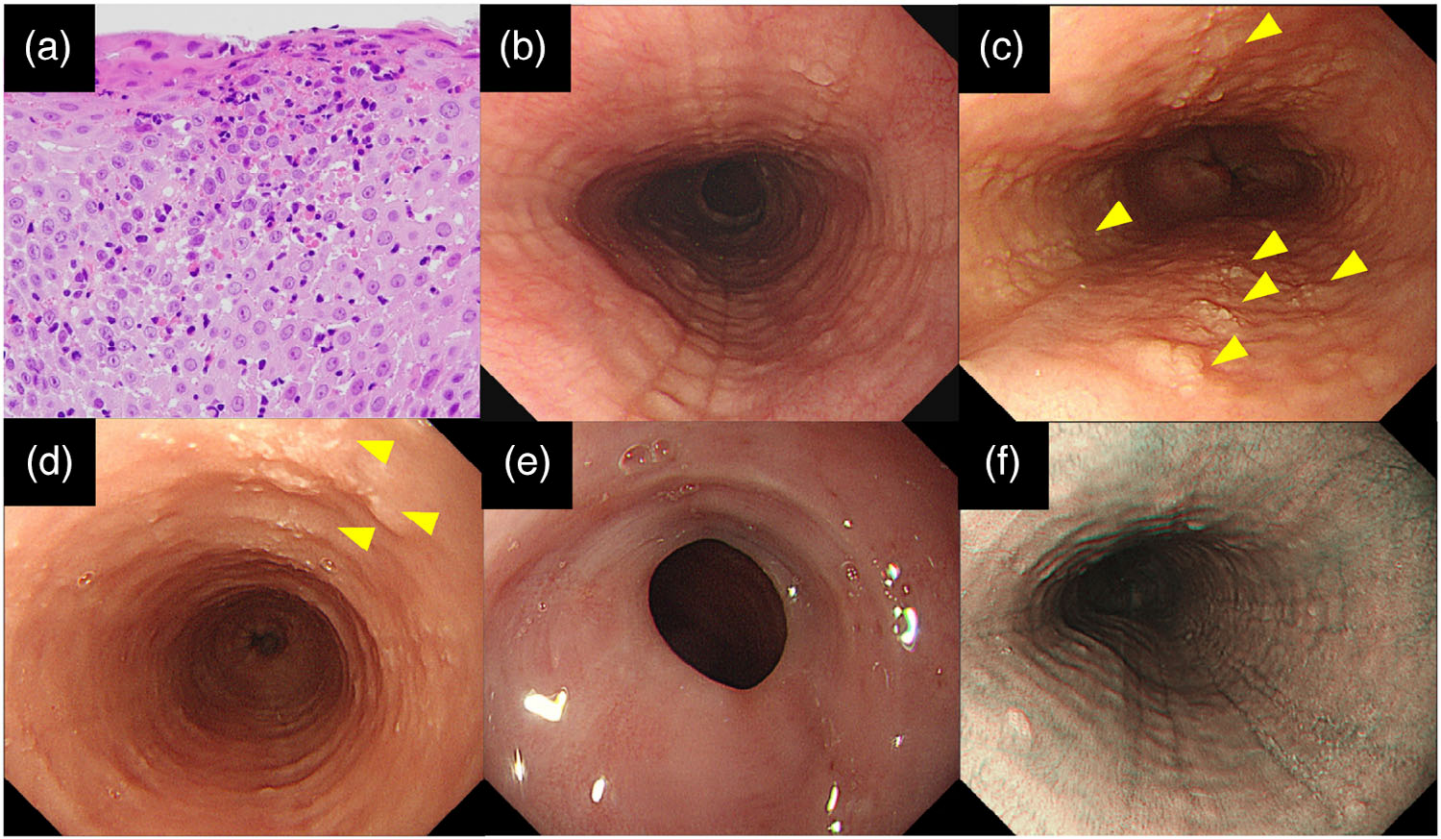
(a) Pathological features of eosinophilic esophagitis. Intraepithelial eosinophilic infiltration (45 eosinophils/high‐power field) and degranulation of eosinophils. (b–f) Endoscopic features of eosinophilic esophagitis; (b) rings, grade 1; furrows, grade 1. (c) edema, grade 1; rings, grade 1; exudates, grade 1; furrows, grade 1. The arrowhead indicates exudate. (d) edema, grade 1; rings, grade 2; exudates, grade 1. (e) strictures, grade 1. (f) Beige mucosa on narrow‐band imaging.

Eosinophilic esophagitis (EoE) is a chronic allergic disease and type 2 helper T cell (Th2)‐mediated immune reactions to food and aeroallergens are involved in its pathophysiology.^1^ EoE is characterized by intraepithelial infiltration of ≥15 eosinophils per high‐power field (HPF) and clinical symptoms of esophageal dysfunction, such as dysphagia, food impaction, chest pain, and reflux symptoms (Figure 1a).^2^, ^3^ The incidence of EoE has increased rapidly and currently varies widely from 1 to 20 new cases per 100,000 inhabitants per year in Western countries.^2^ The prevalence of EoE has also increased, ranging from 13 to 49 cases per 100,000 inhabitants.^2^, ^4^ Although EoE remains less common in Japan than in Western countries, similar to Western countries, the incidence and prevalence of EoE has steadily increased in Japan over 2 decades.^5^ The incidence and prevalence of EoE in Japan were 2.82 per 100,000 person‐years and 10.68 per 100,000 people in 2022, nearly three and eight times higher than in 2017, respectively.

The cause of the increasing incidence may be not only increased sensitization to allergens but also increased awareness of EoE and adequate performance of diagnostic methods. In particular, an endoscopic approach, including esophageal biopsy and eosinophil counting, is essential for the diagnosis of EoE. Endoscopic intervention is a therapeutic approach for patients with severe esophageal strictures. In this review, we describe the endoscopic approach and management of EoE.

### Endoscopic characteristics of EoE

Esophagogastroduodenoscopy (EGD) is an easy and reliable method of diagnosing and managing patients with EoE. The EoE endoscopic reference score (EREFS) is commonly used as a standardized tool to classify and grade the presence and severity of five representative endoscopic esophageal features of EoE (Table 1).^6^ EREFS is an acronym for the five major endoscopic features: edema, rings, exudates, furrows, and strictures. Representative features include edema (or decreased vascularity), rings (also called trachealization), white exudates (also referred to as plaques or spots), longitudinal furrows, and strictures (Figure 1b–e). In the EREFS system, the sum score ranges from 0 to 8. Inflammatory activity is demonstrated by the presence of edema, exudates, and furrows, whereas fibrostenotic remodeling was evaluated by the presence of rings and strictures. The sum of the inflammatory score ranges from 0 to 4, and the sum of fibrostenotic scores ranges from 0 to 4.

**TABLE 1:** The endoscopic reference score system for eosinophilic esophagitis.

• Edema
Grade 0: absent (distinct vascularity present)
Grade 1: loss of clarity or absence of vascular markings
• Rings
Grade 0: none
Grade 1: mild (subtle circumferential rings)
Grade 2: moderate (distinct rings that do not impair the passage of a standard diagnostic endoscope (outer diameter 8–9.5 mm)
Grade 3: severe (distinct rings that do not permit the passage of a diagnostic endoscope)
• Exudates
Grade 0: none
Grade 1: mild (lesions involving <10% of esophageal surface area)
Grade 2: severe (lesions involving >10% of esophageal surface area)
• Furrows
Grade 0: absent
Grade 1: present
• Strictures
Grade 0: absent
Grade 1: present

The EREFS classification system is useful for identifying adult and pediatric patients with EoE.^7^, ^8^ A study of American adult participants showed an area under the receiver operating characteristic curve of 0.934, sensitivity of 88%, specificity of 92%, and accuracy of 91%.^6^ Using the EREFS system, good inter‐ and intraobserver agreements were reported, except for edema, and these were not different between expert and trainee endoscopists.^9^ The EREFS system has been validated as a measure of treatment efficacy, and the core outcome set for therapeutic studies in EoE, including the EREFS system, was developed as a global consensus.^10^ Furthermore, the guidelines for the endoscopic approach to EoE were published at the American Society of Gastrointestinal Endoscopy consensus conference in 2022.^11^

In these guidelines, the EREFS system has been recommended for routine use in the assessment of EoE activity during endoscopy. The disease activity of EoE sometimes differs between locations in the esophagus, such as the proximal and distal esophagus. Regarding the application of the EREFS, the most affected area should be scored as the highest score.^10^ The positive correlation between the EREFS score and mucosal eosinophilia was consistent. As with other endoscopic findings of EoE, beige mucosa on narrow‐band imaging indicated active inflammatory sites (Figure 1f).^12^ The reason for the beige mucosa is the thinning of the superficial squamous cell layer, which allows the blue bands (wavelength 415 nm) to be absorbed by hemoglobin components through the surface of the epithelium.

### Optimal biopsy protocol for the diagnosis of EoE

Knowing the optimal biopsy protocol is quite essential for the assessment of histological activity of EoE. A summary of the optimal biopsy protocols is shown in Table 2. The distribution of esophageal eosinophilic infiltration is patchy and varies among the esophageal locations. It also differed between areas with positive and negative endoscopic findings consistent with EoE. Regarding the location, at least six biopsies should be performed to diagnose EoE.^11^ Several studies have investigated the optimal number of biopsy samples required to increase diagnostic sensitivity. In a series of 102 biopsies from the mid and distal esophagus, the probabilities of one, four, five, and six biopsy samples containing >15 eosinophils/HPF were 0.63, 0.98, 0.99, and >0.99, respectively.^13^ In clinical practice, taking six biopsy specimens is sometimes difficult, especially at medical health check‐ups due to institutional reasons. Therefore, we have to pay attention to the possibility of underdiagnosis for EoE if we obtain <6 biopsy specimens. Moreover, for accurate diagnosis of EoE, anti‐acid drugs such as proton pump inhibitors (PPI) should be withdrawn at least 2 weeks prior to endoscopy and biopsy.

**TABLE 2:** The summary of optimal biopsy protocol.

• At least 6 biopsy samples should be taken to diagnose eosinophilic esophagitis (EoE).
• Anti‐acid drugs such as proton pump inhibitors (PPI) should be withdrawn at least 2 weeks prior to endoscopy and biopsy.
• Biopsies from different locations, such as the distal and proximal/mid esophagus (e.g., two from the distal, two from the mid, and two from the proximal esophagus) are important.
• Obtain a target biopsy from the area with exudates, which is mostly correlated with eosinophilic infiltration.
• If EoE is suspected, a biopsy should be performed even if the esophagus appears normal.
• If patients with EoE have gastrointestinal symptoms that may be derived from non‐EoE eosinophilic gastrointestinal diseases, biopsies of the stomach and duodenum should be performed using esophagogastroduodenoscopy (EGD).
• To evaluate the therapeutic response, EGD with biopsy should be considered at 8 or 12 weeks after pharmacological and dietary treatment.
• The assessment of inflammatory features such as edema, exudates, and furrows may be an ideal endpoint for evaluating the therapeutic efficacy of pharmacological and dietary treatments.

Another important point of view is obtaining biopsies from different locations, such as the distal and proximal/mid esophagus.^11^ The pathophysiology of EoE sometimes overlaps with that of gastroesophageal reflux disease. Because the distal esophagus is the area most affected by gastroesophageal reflux disease, a biopsy of the distal esophagus is reasonable. However, it is unclear whether the proximal or mid‐esophagus is superior in diagnosing EoE. Biopsy samples taken from just below the upper esophageal sphincter are unlikely to demonstrate esophageal eosinophilia,^14^ therefore those from the mid‐esophagus may be a better choice. At our institute, we routinely perform six biopsies, including two from the distal esophagus, two from the mid‐esophagus, and two from the proximal esophagus. Among the inflammatory features of the EREFS system, exudates are most positively correlated with eosinophilic infiltration.^7^, ^8^ Therefore, it is important to perform a targeted biopsy of the area containing exudates.

Although most patients with EoE have abnormal endoscopic findings compatible with EoE, they sometimes appear normal on EGD. Normal endoscopic findings are observed in 10%–32% of both adult and pediatric patients with EoE.^15^, ^16^ Accordingly if EoE is suspected, a biopsy should be performed to diagnose EoE, even if the esophagus appears normal. However, the wide range in the ratio of normal appearance may depend on the proficiency of endoscopists in diagnosing EoE. It is possible that EoE is underdiagnosed when non‐experts perform EGD.

Patients with non‐EoE eosinophilic gastrointestinal diseases (EGIDs) sometimes have concomitant EoE. The nomenclature of whether this phenomenon is called ‘Non‐EoE EGIDs and EoE’ or ‘Non‐EoE EGIDs and esophageal involvement (EI)’ has not yet reached a consensus.^17^ In a study using the US National Administrative Database, the prevalence of concomitant EoE was 10.6% for eosinophilic gastritis (EoG), 12.0% for eosinophilic gastroenteritis (EGE), and 10.9% for eosinophilic colitis (EoC) in the study.^18^

Sato et al. reported the comparison of clinical characteristics, endoscopic and histologic findings, and molecular profiles between isolated EoE and EI with non‐EoE EGIDs.^19^ Male predominance was similarly observed in EoE and EI (76.5% vs. 77.9%). Patients with EoE or EI had similar concomitance of atopic conditions, although patients with EI were more likely to have eczema and food allergy. Abdominal pain was more prevalent in patients with EI than EoE, while other symptoms and endoscopic findings were not different between the groups. Peak esophageal eosinophil count in patients with EI was significantly higher than that in patients with EoE (115.0 vs. 92.0 eosinophils/HPF). Peripheral blood eosinophil levels were also significantly higher in patients with EI than EoE (0.44 × 10^3^/µL [6.0%] vs. 0.38 × 10^3^/µL [5.0%]). Interestingly, EoE and EI were not significantly distinguishable by transcriptome analysis of esophageal biopsy samples. These results indicate that gene expression profiles may be specific for affected organs regardless of other gastrointestinal involvement, and EoE and EI may have shared pathogenesis.

In contrast, if patients with EoE have other symptoms not derived from EoE, such as nausea, vomiting, abdominal pain, and diarrhea, multiple biopsies should be considered from the stomach and duodenum during the initial or subsequent EGD to diagnose non‐EoE EGIDs. The classification system for typical endoscopic findings of EoG is the eosinophilic gastritis endoscopic reference system (EG‐REFS) proposed by the Consortium of Eosinophilic Gastrointestinal Disease Researchers, similar to the EREFS for EoE.^20^ In the 98 patients with EoG defined by a threshold of ≥ 30 eosinophils/HPF, the most common findings were erythema (72%), raised erosion (49%), erosion (46%), and granularity (35%). The EG‐REFS score was significantly associated with histological activity. In Japan, of the 18 patients with EoG or EGE defined by a threshold of ≥ 20 eosinophils/HPF, erythema was most frequently observed (72%), followed by ulcers (39%), discoloration (33%), erosions (28%), nodularity (28%), and polyps (28%) in the stomach.^21^ A normal endoscopic appearance in patients with non‐EoE EGIDs may be common; however, the range was wide, from 8% to 80%, which may depend on the involved organ, the threshold of eosinophilic infiltration, and the number of participants.^18^ Taken together, taking biopsies from not only abnormal findings but also the normal appearance of the stomach and duodenum should be considered on EGD diagnosing concomitant EoG and EoD when patients with EoE have extraesophageal gastrointestinal symptoms.

### Assessment of response to therapy

To evaluate the efficacy of pharmacological treatments and dietary elimination therapy, an assessment of endoscopic and histologic findings, including eosinophilic infiltration, as well as symptoms, is needed. In addition to symptomatic relief, the normalization of endoscopic features, especially inflammatory findings and decreased eosinophilic infiltration below the diagnostic threshold, are therapeutic goals. Endoscopy with biopsy should be considered at 8 or 12 weeks after treatment because several clinical studies have set the endpoint at that time.^22^, ^23^, ^24^, ^25^

The EREFS system is a standardized, easy‐to‐use method for evaluating endoscopic improvement. However, there is some caution regarding the use and interpretation of the EREFS system because the endoscopic findings of the EREFS system are not completely specific to EoE and could be found in other esophageal disorders, such as gastroesophageal reflux disease. The area under the curve to determine the response to treatment ranged from 0.79^26^ to 0.88,^8^ indicating that 12%–21% of the patients would be misevaluated. Therefore, improvement in inflammatory features, such as edema, exudates, and furrows, may be an ideal endpoint for evaluating the pharmacological and dietary therapeutic efficacy of EoE.^11^ Pharmacological treatments, such as PPI, swallowed topical corticosteroids, and biologics, are anti‐inflammatory agents that lead to reduced eosinophil infiltration, and dietary elimination therapy leads to a reduced inflammatory response to food allergens. Fibrostenotic features, such as rings and strictures, were sometimes fixed as a consequence of remodeling, and these features were difficult to improve with anti‐inflammatory therapy. Accordingly, inflammatory features should be considered separately from fibrostenotic features when evaluating the efficacy of pharmacological and dietary therapies.

Regarding the biopsy protocol, several biopsies are needed to evaluate the therapeutic efficacy similar to the diagnosis. In 22 PPI nonresponders with 124 biopsies, the prevalence of esophageal eosinophilia was 76.2, 40.9, and 24.3% in the distal, mid, and proximal esophagus, respectively.^27^ The positive predictive value for the non‐effectiveness of PPI was 0.910 in cases with four biopsies taken from the distal and mid‐esophagus; therefore, at least four biopsies are needed. Since there is sometimes a discrepancy between symptomatic relief and histological remission, evaluating histological activity is an important objective measure. In clinical trials, 0–1 eosinophils/HPF was considered histologic complete remission, and 2–14 eosinophils/HPF was considered histologic partial remission.^24^, ^28^ We previously reported that esophageal mast cells might be involved in the mechanism of symptomatic perception in EoE^29^; however, there are no established methods for evaluating the number and activity of esophageal mast cells in clinical practice.

### The index of severity for EoE

In 2022, the Index of Severity for EoE (I‐SEE) was newly developed during a consensus conference facilitated by the American Gastroenterological Association and with the participation of a multidisciplinary team to identify key features of disease activity that were meaningful to clinicians and patients.^30^ The I‐SEE tool included three domains: symptoms and clinical complications; inflammatory features; and fibrostenotic features (Table 3). Scoring was 0 for inactive, 1–6 for mild, 7–14 for moderate, and ≥ 15 for severe EoE. In the post‐hoc analysis of randomized controlled study, I‐SEE correlated with clinical features at diagnosis, and severity improvement with successful topical corticosteroid treatment.^31^ The usefulness of I‐SEE was also shown in the assessment of severity for the children treated long‐term during routine clinical care.^32^

**TABLE 3:** The index of severity for eosinophilic esophagitis.

Points per feature	1 point	2 points	4 points	15 points
**Symptoms and complications**				
Symptoms	Weekly	Daily	Multiple times per day or disrupting social functioning	–
Complications	–	Food impaction with emergency room visit or endoscopy (*patient ≥ 18 years*)	·Food impaction with emergency room visit or endoscopy (*patient < 18 years*) ·Hospitalization due to eosinophilic esophagitis	·Esophageal perforation ·Malnutrition with body mass < 5th percentile or decreased growth trajectory ·Persistent inflammation requiring elemental formula, systemic corticosteroid, or immunomodulatory treatments
**Inflammatory features**				
Endoscopy (edema, furrows, and/or exudates)	Localized	Diffuse	–	–
Histology	15–60 eosinophils /high‐power field	>60 eosinophils /high‐power field	–	–
**Fibrostenotic features**				
Endoscopy (rings, strictures)	Present, but endoscope passes easily	Present, but requires dilation or a snug fit when passing a standard endoscope	–	Cannot pass standard upper endoscope; repeated dilations (*in an adult ≥ 18 years*); or any dilation (*in a child < 18 years*)
Histology	–	Basal zone hyperplasia or lamina propria fibrosis (or dyskeratotic epithelial cells /surface epithelial alteration if no lamina propria)	–	–

### Asymptomatic esophageal eosinophilia

We occasionally encounter asymptomatic esophageal eosinophilia (aEE) on EGD mainly during medical health check‐ups. We previously reported that the prevalence of EoE and aEE during medical health check‐ups in 2019 was 0.34% (20/5964 persons) and 0.12% (seven persons), respectively.^33^ The endoscopic and histologic findings and immune profile were similar between EoE and aEE; therefore, these diseases may be the same disease entity.^34^, ^35^ In a retrospective study that observed the natural history of aEE, of 21 patients with aEE who did not have any medications, endoscopic findings worsened in two (9.5%), improved spontaneously in seven (33.3%), unchanged in 12 (57.1%), and three patients (14.3%) developed symptoms at a mean time of 40 months during the median follow‐up time of 64 months.^36^ Accordingly, it is important to monitor disease activity regularly by EGD and take tailored treatment based on the needs of each patient.

### Treatment for EoE

#### PPI and potassium‐competitive acid blockers

**FIGURE 2:**
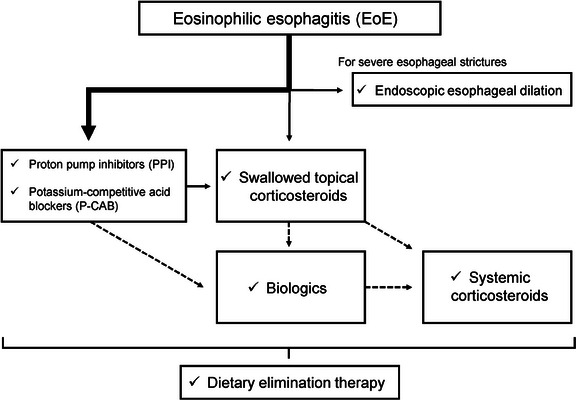
Flow chart of the treatment for eosinophilic esophagitis. Proton pump inhibitors (PPI) and potassium‐competitive acid blockers (P‐CAB) are most commonly used as first‐line therapies. Swallowed topical corticosteroids and biologics are approved in Western countries. Food elimination can be added to pharmacological therapy.

A flowchart of the EoE treatment is shown in Figure 2. As a medical therapy for EoE, anti‐acid drugs such as PPI and potassium‐competitive acid blockers (P‐CAB) are most commonly used as first‐line therapies. In a real‐world data analysis of 15,200,895 individuals and 615 patients with EoE using an employer‐based insurance claims database in Japan, PPI/P‐CAB accounted for 80% of first‐line therapy.^37^ Real‐world data from 613 patients with EoE in the United States demonstrated that PPI accounted for 51% of the initial therapy.^38^ In a retrospective study of 236 patients with EoE who received first‐line treatment with PPI or P‐CABs for 8 weeks, complete normalization was achieved in 25%, 50%, 36%, and 8% of the patients for symptoms, endoscopy (EREFS score 0), histology (0–1 eosinophil/HPF), and all three outcomes, respectively.^25^ Furthermore, the response rates were 81%, 87%, 75%, and 60% for symptoms, endoscopy (EREFS score ≤ 2), histology (<15 eosinophils/HPF), and all three outcomes, respectively. Although complete normalization was uncommon, more than 75% of patients showed some response.

In a retrospective study, P‐CAB showed an efficacy similar to that of PPIs with regard to symptomatic, endoscopic, and histologic responses.^24^ The complete symptomatic relief rates were 54.5% for rabeprazole 10 mg/day, 64.7% for rabeprazole 20 mg/day, 72.0% for esomeprazole 20 mg/day, and 75.7% for vonoprazan 20 mg/day. Although attention should be paid to this interpretation because of the retrospective nature of the study, these results may indicate the limitations of anti‐acid drugs in improving the symptoms of EoE. In the systematic review and meta‐analysis of 619 patients, although heterogeneity was high, the rate of histologic remission (<15 eosinophils/HPF) for PPI was 50.5% (95% confidence interval [CI]: 42.2–58.7) and the clinical response rate was 60.8% (95% CI: 48.4–72.2).^39^ In previous retrospective and prospective studies, the doses and therapeutic periods of PPI varied. To date, no randomized, placebo‐controlled study has evaluated the efficacy of PPI. In the UK guidelines, PPI treatment for at least 8–12 weeks is recommended prior to histological assessment.^40^

Regarding maintenance therapy, in a real‐world analysis in Japan, PPI/P‐CAB use rapidly decreased by 40% in the first 6 months, and afterward, reinitiation was rarely seen.^37^ The median time to treatment discontinuation was 172 days (95% CI: 147–206 days). Only 1 patient developed esophageal fibrostenotic complications, such as esophageal foreign bodies, after diagnosis during a median follow‐up time of 700 days. Given the low incidence of fibrostenotic complications, universal maintenance therapy may not be needed for patients with milder disease in Japan than in Western countries.^41^ In contrast, in Western countries, maintenance PPI therapy can be considered a long‐term treatment because of the high risk of relapse after discontinuation of PPI.^2^, ^40^ When pharmacological therapy is discontinued, symptoms and/or esophageal eosinophilia typically recur over 3–6 months.^42^ However, the evidence level for maintenance therapy with PPI is low because there have been no studies beyond 12 months of maintenance therapy. In a prospective study of 57 children administered maintenance PPI therapy (esomeprazole 1 mg/kg/day) for 12 months, the long‐term histological remission rate was 70.1%.^43^ Furthermore, the optimal dosage for the maintenance of PPI therapy and therapeutic strategies is yet to be defined. Therefore, a decrease in the minimum PPI dose that can maintain remission is reasonable.

#### Swallowed topical corticosteroids

Swallowed topical corticosteroids are highly effective in inducing the remission of EoE, with a high evidence level.^2^, ^40^, ^44^ In adult EoE, the budesonide orodispersible tablet (BOT; 1 mg twice daily) was approved in Europe and Australia from 2018 to 2022 and is recommended for use in the guideline.^40^ The usual duration of induction treatment is 6 weeks; however, the duration can be extended up to 12 weeks for patients with an inadequate response at 6 weeks. In a double‐blind, placebo‐controlled study, 58% of patients who were administered BOT for 6 weeks were in complete clinico‐histologic remission compared to those patients given a placebo.^45^ At 6 weeks, histologic remission (<16 eosinophils/HPF) was achieved in 93% of patients administered BOT compared to no patients administered the placebo. At 12 weeks, 85% of the patients who received BOT achieved clinico‐histologic remission. Six‐ and 12‐week BOT therapies were safe and well tolerated; 5% of patients developed symptomatic mild esophageal candidiasis, which was easily treated with an oral antifungal agent.

Regarding maintenance treatment, BOT (0.5 mg or 1 mg twice daily) was also approved, and the dosage was dependent on the clinical requirements of the patients. Because clinical and histologic relapses are high after the withdrawal of topical corticosteroids, maintenance therapy is recommended in the guidelines.^40^, ^44^ Moreover, in 2024, 12 weeks of treatment with a novel formulation of budesonide oral suspension (2 mg twice daily) was approved in the United States for patients aged 11 years and older with EoE.

In a real‐world Japanese analysis, swallowed topical corticosteroids were administered to 4.6% of patients with EoE as first‐line therapies.^37^ The proportion of swallowed topical corticosteroids reduced to 2% at 24 months. The median time to discontinuation of swallowed topical corticosteroids was 43 days, which was shorter than that of PPI/P‐CAB. Swallowed topical corticosteroids are not approved by Japanese insurance, and swallowing of corticosteroids for inhaled use was commonly performed. For PPI/P‐CAB‐refractory patients, switching from PPI/P‐CAB to swallowed topical corticosteroids is a reasonable option; however, the rate of this switching in clinical practice was low (2% in the first 6 months and 1.6% in the second 6 months).

Systemic corticosteroids are not recommended in the guidelines.^40^ In a pediatric randomized controlled trial, the histological response rate at 4 weeks was not different between prednisolone (1 mg/kg, 2 times per day) and swallowed fluticasone (220 mg or 440 mg four times per day according to age); however, adverse events were more frequently observed in the prednisolone group (40%: hyperphagia, weight gain, and Cushingoid appearance).^46^ Systemic corticosteroids may be the final pharmacological option for managing adult patients with EoE refractory to other medications; however, evidence is lacking.

#### Biologics

Biologics represent a new therapeutic option for inhibiting the action of Th2‐mediated cytokines. Dupilumab was the first biologic drug approved by the U.S. Food and Drug Administration in 2022 for patients with EoE aged 12 years and older. In 2024, dupilumab has also been approved for the treatment of pediatric patients aged 1–11 years. Dupilumab is a fully human monoclonal anti‐interleukin (IL)‐4Rα antibody that blocks both IL‐4 and IL‐13 because IL‐4Rα is a shared receptor. In a phase 3 trial, the rate of histologic remission with dupilumab at a weekly dose of 300 mg was significantly higher than that with placebo at 24 weeks (60% vs. 5%).^47^ The use of dupilumab has also been approved for other allergic diseases, such as bronchial asthma, chronic rhinosinusitis with nasal polyps, atopic dermatitis, and chronic spontaneous urticaria.^48^, ^49^, ^50^, ^51^ Therefore, patients with EoE and other allergic comorbidities may benefit from dupilumab treatment.

Cendakimab, a monoclonal anti‐IL‐13 antibody, significantly reduced histological and endoscopic activity in a phase 2 trial.^52^ Biologics targeting eosinophils via IL‐5, such as mepolizumab, reslizumab, and benralizumab, have been tested in randomized controlled trials. These biologics induce histological remission; however, they fail to improve the symptoms.^53^, ^54^, ^55^ These results indicated that targeting eosinophils is not sufficient to improve symptoms.

#### Dietary elimination therapy

Food elimination is an important strategy for reducing food antigens that cause and exacerbate allergic reactions to EoE. Food elimination can be added to pharmacological therapy and, if effective, medications can be reduced. Food allergy tests, such as the skin prick test, patch test, and serum food‐specific immunoglobulin E measurement, are not informative for detecting antigen triggers.^56^, ^57^ A six‐food elimination diet (6FED) is used to eliminate major 6 allergens (dairy, wheat, egg, soy, seafood, and nuts). It is effective and is commonly used in clinical practice; however, it is poorly tolerated, especially by adults.^58^ Recently, in a randomized open‐label trial, eliminating animal milk alone (1FED) had a similar efficacy in histological and endoscopic improvement as 6FED.^59^ The 6FED regimen was effective in less than half of the 1FED non‐responders. 1FED is acceptable and easier to perform than 6FED, and 1FED is expected to become a new elimination therapy.

#### Endoscopic esophageal dilation

Endoscopic esophageal dilation is a therapeutic option for fibrotic strictures due to EoE to improve obstructive symptoms, such as dysphagia and food impaction. The precise prevalence of esophageal stricture in Japan is unclear because a large‐scale epidemiological study was not conducted to observe this point of view. Moreover, whether the cases of esophageal stricture are increased has not been elucidated. In the systematic review, Kinoshita et al. reported esophageal stricture was rare in 188 Asian patients with EoE, and the prevalence of patients with food impaction was 4.2% (9/213 cases).^60^ Because Japanese EoE is milder than in Western countries.^41^ the need for endoscopic esophageal dilation is less common.^61^

The effectiveness and safety of endoscopic dilation were shown in a systematic review of 27 studies with 845 patients who underwent 1820 endoscopic dilations.^62^ Clinical improvement occurred in 95% of patients, and a low rate of major complications was observed: perforation occurred in 0.38%, hemorrhage in 0.05%, and hospitalization in 0.67%. In a systematic review of 977 patients with 2034 dilations, no difference in perforation risk was reported between dilator types, such as bougie and balloon.^63^ In a retrospective study of 66 patients with severe stricture, defined as an esophageal diameter < 10 mm, medical or dietary therapy in combination with repeated esophageal dilation achieved a diameter of ≥ 13 mm in 89% of patients and a diameter of ≥ 15 mm in 65% of patients.^64^ The initial diameter and histological remission were significantly associated with achieving a diameter of ≥ 15 mm.

## CONCLUSIONS

We reviewed the endoscopic management to diagnose and evaluate the inflammatory activity of EoE and therapeutic options. We hope that this article will help doctors adequately manage patients with EoE in clinical practice.

## CONFLICT OF INTEREST STATEMENT

None.
